# From a Philosophical Framework to a Valid Prognostic Staging System of the New “Comprehensive Assessment” for Transplantable Hepatocellular Carcinoma

**DOI:** 10.3390/cancers11060741

**Published:** 2019-05-28

**Authors:** Stefano Di Sandro, Vincenzo Bagnardi, Alessandro Cucchetti, Andrea Lauterio, Riccardo De Carlis, Laura Benuzzi, Maria Danieli, Francesca Botta, Leonardo Centonze, Marc Najjar, Luciano De Carlis

**Affiliations:** 1 Department of General Surgery and Transplantation, Niguarda Ca’ Granda Hospital, 20162 Milan, Italy; andrea.lauterio@ospedaleniguarda.it (A.L.); riccardo.decarlis@ospedaleniguarda.it (R.D.C.); laura.benuzzi@ospedaleniguarda.it (L.B.); maria.danieli@ospedaleniguarda.it (M.D.); leonardo.centonze@ospedaleniguarda.it (L.C.); luciano.decarlis@ospedaleniguarda.it (L.D.C.); 2 Niguarda Transplant Foundation, Niguarda Ca’ Granda Hospital, 20162 Milan, Italy; vincenzo.bagnardi@unimib.it; 3 Department of Statistics and Quantitative Methods, University of Milan-Bicocca, 20162 Milan, Italy; f.botta1@campus.unimib.it; 4 Department of Medical and Surgical Sciences—DIMEC; Alma Mater Studiorum, University of Bologna, 40126 Bologna, Italy; aleqko@libero.it; 5 Morgagni–Pierantoni Hospital, 47121 Forli, Italy; 6 Department of Surgical Sciences, University of Pavia, 27100 Pavia, Italy; 7 Center for Liver Disease and Transplantation, Columbia University Medical Center, New York Presbyterian Hospital, New York, NY 10032, USA; mn2594@cumc.columbia.edu; 8 School of Medicine and Surgery, University of Milano-Bicocca, 20162 Milan, Italy

**Keywords:** liver transplantation, hepatocellular carcinoma, transplantable tumor

## Abstract

The comprehensive assessment of the transplantable tumor (TT) proposed and included in the last Italian consensus meeting still deserve validation. All consecutive patients with hepatocellular carcinoma (HCC) listed for liver transplant (LT) between January 2005 and December 2015 were post-hoc classified by the tumor/patient stage as assessed at the last re-staging-time (ReS-time) before LT as follow: high-risk-class (HRC) = stages TT_DR_, TT_PR_; intermediate-risk-class (IRC) = TT0_NT_, TT_FR_, TT_UT_; low-risk-class (LRC) = TT1, TT0_L_, TT0_C_. Of 376 candidates, 330 received LT and 46 dropped-out. Transplanted patients were: HRC for 159 (48.2%); IRC for 63 (19.0%); LRC for 108 (32.7%). Cumulative incidence function (CIF) of tumor recurrence after LT was 21%, 12%, and 8% at 5-years and 27%, 15%, and 12% at 10-years respectively for HRC, IRC, and LRC (*P* = 0.011). IRC patients had significantly lower CIF of recurrence after LT if transplanted >2-months from ReS-time (28% vs. 3% for <2 and >2 months, *P* = 0.031). HRC patients had significantly lower CIF of recurrence after-LT if transplanted <2 months from the ReS-time (10% vs. 33% for <2 and >2 months, *P* = 0.006). The proposed TT staging system can adequately describe the post-LT recurrence, especially in the LRC and HRC patients. The intermediate-risk-class needs to be better defined and further studies on its ability in defining intention-to-treat survival (ITT) and drop-out are required.

## 1. Introduction

Over the past twenty years, physicians’ belief in the curative potential of liver transplantation (LT) for early hepatocellular carcinoma (HCC) on liver cirrhosis has been strongly reinforced, particularly in centers where LT is an available alternative of cure [1,2,3,4,5]. The choice of the best candidates and the decision of whether to list a patient for transplantation is currently a field of research worldwide. Patients are currently selected and prioritized on specific and validated criteria (Milan Criteria, BCLC criteria, UCSF criteria, etc.) [5,6,7]. Before eventual LT, potential candidates are treated with various neo-adjuvant therapies to either downsize tumors or to decrease the risk of tumor progression and recurrence [8].

Three main concepts may guide the philosophy of listing and assigning priority for a patient waiting for LT: utility, urgency, and survival benefit. Accordingly, several models have been proposed to date [9,10,11,12,13]. However, it should be noted that relying solely on tumor burden and/or alpha-fetoprotein (AFP) cannot fully figure the biological aggressiveness of HCCs. That is, patients with the same tumor stage may have a different risk of dropout from the waiting list or of tumor recurrence after transplant. This could well be the consequence of both the possibility of offering patients neo-adjuvant surgical and loco-regional treatments (LRTs) and on the efficacy of these treatments [8,14].

Recently, a comprehensive assessment of patients affected by HCC and suitable for transplant was proposed by Mazzaferro V. [15] and subsequently included into the Italian Consensus-Based Approach to Organ Allocation in Liver Transplantation [16]. Such a concept overcomes the limits of various staging systems published to date, by simply defining HCCs as transplantable or not. Eight classes of transplantable tumor (TT) were identified through the combination of tumor stage, suitability for LRTs, and tumor response to those treatments. To date, despite such conceptual system was incorporated within Italian consensus, its validation has never been assessed whereas it should be fundamental when developing recommendations.

The aims of the present study were to apply such a TT staging to evaluate post-LT HCC recurrence and survival, as well as to evaluate its suitability in describing different probabilities of drop-out. With these aims, the present study constitutes the first effort of validation of the proposed assessment which could be useful to identify those candidates who deserve prioritization for LT and within which time-frame.

## 2. Results

**Figure 1:**
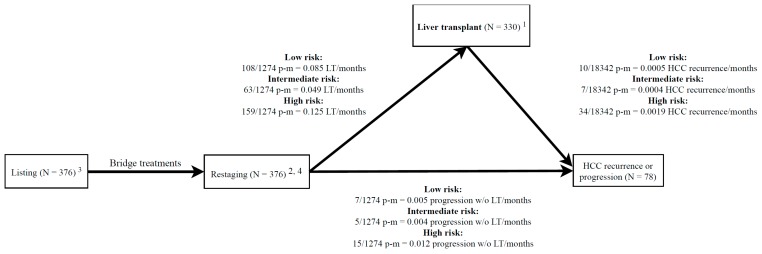
Management of patients with hepatocellular carcinoma (HCC) from listing to HCC relapse in a multi-state framework. The lines connecting the boxes (states) represent the four transitions of interest (i.e., from listing to restaging, from restaging to liver transplant, from restaging to HCC progression w/o liver transplant, and from liver transplant to HCC recurrence). ^1^ The date of liver transplant is the starting time for the estimation of cumulative incidence function (CIF) of HCC recurrence after liver transplant (LT) (Figure 2, Figure 4). ^2^ The date of restaging is the starting time for the estimation of CIF of LT (Figure 3A) or HCC progression without LT (Figure 3B). ^3^ The date of listing is the starting time for the estimation of intention-to-treat survival function (Figure S1A) and CIF of HCC recurrence or progression (Figure S1B). ^4^ *N* = 19 patients dropped-out for other causes than HCC progression (19/1274 p-m (person-month) = 0.015 ways out from list/months).

From January 2005 to December 2015, 376 patients were candidates for LT because of HCC on liver cirrhosis (Table 1). Figure 1 reports the patients’ course from the time of listing, through re-staging (ReS-time) to LT or dropout. Of these, 330 were transplanted and 46 patients dropped-out because of tumor progression (27 cases) or other reasons (19 cases) during follow-up.

### 2.1. Impact of Re-Staging Features on Post LT Outcome

The 330 transplanted patients were classified at the ReS-time into: HRC for 159 (48.2%); IRC for 63 (19.1%); and LRC for 108 (32.7%). Characteristics of transplanted patients are detailed in the Table S1. In summary, at listing the IRC candidates had higher number of HCCs and the LRC had smaller tumors, so that the final T-stage at listing resulted more advanced in the IRC group than the HRC and LRC (*P* < 0.0001). Intermediate-risk class patients had more compromised liver function, as depicted by higher MELD score. At ReS-time, HRC had more advanced tumors, as excepted. Finally, the pathological evaluation of the explanted livers revealed more numerous and larger nodules in the IRC and HRC groups (*P* < 0.0001) and the prevalence of G3 tumors and microvascular invasion were significantly higher in the HRC group (*P* < 0.0001 for both).

**Figure 2:**
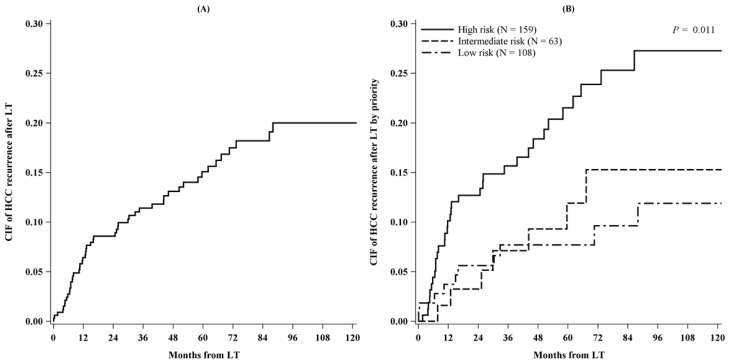
Cumulative incidence of HCC recurrence after liver transplantation overall (**A**) and by priority (**B**) (*N* = 330).

On this ground, 51 tumor recurrences were observed among the 330 transplanted patients. The overall cumulative incidences of tumor recurrence after LT at 5 and 10 years respectively were 15% and 20% (Figure 2A). The 5-year recurrence rates were 21% in HRC, 12% in IRC, and 8% in LRC (Figure 2B; *P* = 0.011). The 5-year overall post-LT survival was 78% (95% CI: 73–82%).

Hepatitis B or Hepatitis C as underlying diseases were not associated to a significant different cumulative HCC recurrence after transplant (Figure S1).

**Figure 3:**
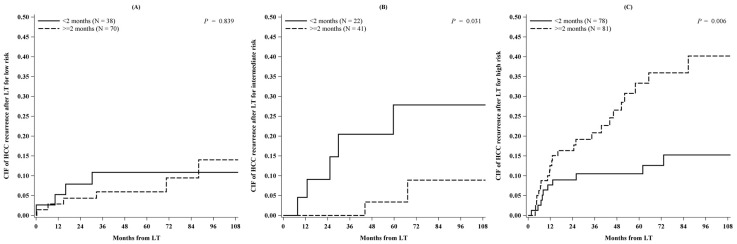
Cumulative incidence of HCC recurrence after liver transplantation for (**A**) low, (**B**) intermediate and (**C**) high risk stratified between transplanted patients within 2 months and after 2 months from restaging (*N* = 330).

Tumor recurrence was found to be related not only to the risk-class at re-staging but also to the time elapsed from re-staging to LT. In particular, IRC patients showed a significantly lower cumulative incidence (95% CI) of 5-year tumor relapse after LT if the interval between times ReS-time and LT-time was ≥2 months (3% (0.2–15%) vs. 28% (9–50%) when <2 months; *P* = 0.031). Conversely, HRC patients had a significantly lower cumulative incidence of 5-year tumor relapse after LT if transplanted within 2 months from the ReS-time (10% (4–19%) vs. 33% (22–45%) when ≥2 months; *P* = 0.006) (Figure 3).

High-risk class and alpha-fetoprotein at the Re-S-time resulted independently related to tumor recurrence after LT: HR = 2.89 (95% CI: 1.26–6.64; *P* = 0.012) and HR = 1.23 (95% CI: 1.10–1.37; *P* = 0.0002), respectively (Table 2). The alpha-fetoprotein level and pathological features at the LT-time were not significantly different among patients transplanted within 2 months or after 2 months from the ReS-time both in the IRC and HRC groups. The only significant different feature was the median diameter of the largest nodule in the IRC group, which was larger among patients transplanted ≥2 months (20 mm vs. 26 mm, *P* = 0.04) (Table 3).

### 2.2. Dropped-Out From WL

A total of 46 patients dropped out in a median time of 209 days (IQR: 104–369 days) from listing and 132 days (IQR: 49–195 days) from ReS-time. Among these, 16 were in LRC (35%), 7 in IRC (15%), and 23 in HRC (50%) at ReS-time. The majority of dropped-out patients had tumor progression (27/46, 59%), whereas 19/46 (41%) patients were excluded from LT for other reasons (Table 1).

### 2.3. Intention-to-Treat and Survival

**Figure 4:**
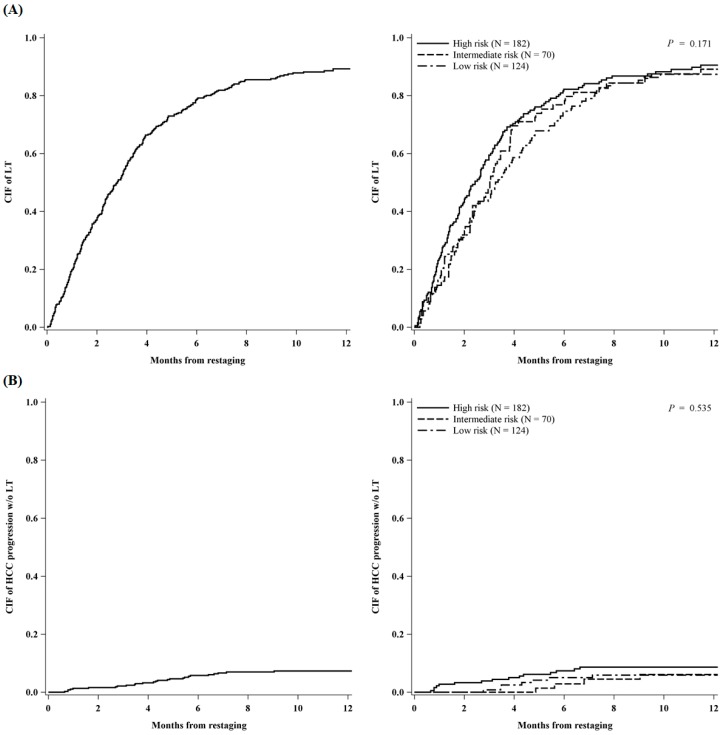
Cumulative incidence of liver transplantation (**A**) and HCC progression w/o liver transplantation (**B**) calculated from restaging, overall and by priority (*N* = 376).

Reconsidering the whole study population of 376 patients, the 5-year intention-to-treat survival (ITTs) was 70% (95% CI: 64–74%), from listing (Figure S2). Since the ReS-time, patients had a cumulative incidence of transplant at 1-year (95% CI) of 89% (85–92%) and a cumulative incidence of tumor progression without LT at 1-year (95% CI) of 7% (5–10%) (Figure 4).

Figure 4 showed no statistically significant difference in the cumulative incidence of LT or tumor progression without transplant among the three groups; however, the curves showed a trend towards higher incidences (of LT and tumor progression) in the higher-risk patients’ class.

## 3. Discussion

To the best of our knowledge, this is the first effort to validate the new comprehensive assessment of transplantable tumor (HCC) adopted in the Italian Consensus-Based new allocation system [16]. The present study consisted of an a posteriori application of the proposed TT staging. The opportunity to validate this new staging on a patient cohort treated before its proposal should reinforce the obtained results. Indeed, although patients were selected for LT and prioritized based on different criteria throughout the study time (2005–2015), we observed a linear trend between the probability of receiving a LT or dropping out secondary to tumor progression and the severity of the TT class. This correlation, however, was not statistically significant.

Patients classified into the HRC group (TT_DR_ and TT_PR_) had higher incidence of both being transplanted and dropping than those in the IRC and LRC groups. Moreover, HRC re-staged patients had a significantly higher risk of tumor recurrence after transplant, particularly if they were transplanted later than 2 months from the ReS-time. Several authors have previously suggested that the worst outcomes are observed when down-staging treatments prior to LT are not fully effective, namely higher incidences of tumor recurrence after transplant or dropouts without transplant [14,17,18]. The HRC group included patients with tumor progression after downstaging (TT_DR_) or patients who had a partial tumor response after LRTs (TT_PR_). Related to these two categories of patients, this study suggested that a high priority to LT (possibly within 2 months from ReS-time) may significantly reduce the risk of tumor recurrence after LT. In other words, the sooner the HRC patients are transplanted, the lower their tumor recurrence rates will be. Although patients in the HRC group received the same LRTs regimens as the other patients, they did not show a complete response to LRTs, this could probably be explained by tumor proper characteristics (i.e., vascularization, location, cell differentiation). However, if these patients were transplanted within 2 months from ReS-time, their tumor cumulative incidence after-LT appeared to be similar to the other groups (<10% at 5 years). We therefore hypothesize that, for these patients a short interval between ReS-time and LT-time may be the key to significantly decrease the rate of post-LT tumor recurrence, by the chance of higher priority or living donor LT.

Surprisingly on the other hand, a too fast-track from ReS-time to LT for patients classified as IRC (TT0_NT_, TT_FR_, and TT_UT_) may be associated with worse tumor-related results. These patients belong to successful downstaging, or HCC > T1 at the first presentation or late recurrent HCC after curative treatments and those patients judged untreatable for reasons not captured by MELD. In the present study the tumor T-stage of IRC patients at the listing appeared to be worse than the other two groups. Despite that, after the successful application of downstaging and bridging treatments, the radiological staging at the ReS-time demonstrated a significant downgrading of the tumor stage for IRC with lower T-stage compared to the HRC group, as expected. However, despite the effective tumor downsizing among these patients, their post-LT 5-year cumulative recurrence rate was up to 28% for those transplanted within 2 months since the ReS-time but, more importantly, 3% for those transplanted later. If compared to findings in the HRC, the present one is not paradoxical as it seems and was simply related to the fact that this risk category had the highest prevalence of untreated candidates: for these cases, the “test-of-time” dominates the clinical outcome. In fact, some authors have recently pointed out the risk of bringing patients too fast to LT [17,18,19,20,21]. Halazun K. and co-workers reported a significantly lower long-term survival among HCC patients transplanted within less than 2 months from the time of listing in the United States [22]. The authors provided evidence that expediting HCC patients to LT at too fast a rate may adversely affect patient outcomes. The adverse effect of the “fast track” was explained by the fact that these patients are more likely to be undertreated before LT as well as by the lack of a test-of-time. The concept of “test-of-time” could be understood as a minimal period between two surveillance time points (imaging, lab values, clinical evaluation) that may help distinguish patients with aggressive diseases that progress quickly, a surrogate marker of an aggressive tumor biology, therefore distinguishing patients who need to be prioritized from those who need to be excluded. In our study, the time interval between the last LRTs and ReS-time was shorter in the IRC compared to the other classes. Although not statistically significant, perhaps IRC patients need a longer interval to better assess the tumor response to LRTs.

A few years ago, several authors pointed out the risk of worse tumor-related results of LT from a living donor (LDLT) than from a deceased one [21,23,24,25]. The proposed hypotheses included: more advanced tumors treated with LDLT and hyper-inflow in a partial transplanted liver as a cancerogenetic promoter. Several authors demonstrated higher tumor recurrences after LDLT, even after adjusting for tumor stage selection before LT [21,23,24,25]. In clinical practice, the availability of a living donor for an HCC patient may discourage physicians from spending time downgrading tumors before transplant; therefore, in some series the rate of patients undergoing LRTs before LDLT is very low while tumor recurrence after LT is high [21,23,24,25]. In that sense, IRC represents a group of patients wherein considering LDLT may be risky due to the probability to go too fast to transplant instead of obtaining a test-of-time for a better assessment of tumor biology. On the other hand, the HRC group may strongly benefit from an early transplant from the ReS-time, an available living donor for those patients may therefore represent the best chance.

The LRC group did not show any correlation with tumor stages and recurrence after LT. Patients included in the LRC group were generally transplanted before the time may adversely affect transplant results without differences among patients transplanted before or after 2 months from ReS-time. The LRC group does not usual pose a crucial management dilemma for physicians, however those patients should be under surveillance according to the international guidelines [19].

The presented results are limited by the retrospective nature of the study. The clinically-based construction of the three risk classes is also limited by the patient cohort size and some of the missing data. The lack of a validation cohort can further weaken our results. We should however hasten to add that the present study was not planned to produce a new prognostic system, rather to verify whether patients can be stratified using the present conceptual framework. As we previously stated, such a framework is currently adopted in the Italian Consensus-Based allocation system and we needed to analytically verify whether corrective maneuvers are necessary. We observed in fact, that IRC patients need to be better identified and we hope that our findings could be taken into account in the future national consensus. In addition, to provide a strong clinical application of the TT system it will be necessary to compare current prognostic ability to that of already developed staging systems in order to find the best tool to adopt. Further studies in this sense represent the next steps since present one.

## 4. Materials and Methods

This is a single-center retrospective study using a prospectively maintained database, including all consecutive patients who were candidate for LT for HCC on liver cirrhosis between January 2005 and December 2015. The Institutional Ethical and Scientific board approved the present study.

Pre-operative diagnosis of HCC was made according to non-invasive European Association for the Study of the Liver (EASL) criteria [4]. In cases lacking conclusive radiological diagnosis, ultrasound-guided biopsy was used [19]. All the cases were discussed in a multidisciplinary meeting with surgeons, hepatologists, radiologists, and anesthesiologists. Patients were followed up for life and received surveillance for the risk of new HCC diagnosis according to the EASL guidelines [19].

Patients were selected for transplant based on the Milan Criteria [6] until 2008 and based on the up-to-seven criteria from 2009 until 2015 [9]. Patients with a tumor stage beyond selection criteria at diagnosis were listed for LT only after an effective down-staging. Indications for down-staging and for HCC bridging treatments during the waiting period to transplantation were previously published [14,26]. All patients treatable by LRTs were treated prior to transplant. Extra-hepatic tumor, macro-vascular invasion, and age >70 years were contraindications for LT in our center. Tumor response to the LRTs was prospectively assessed by the modified RECIST (Response evaluation criteria in solid tumors) criteria since their release, and retrospectively for cases before 2010 [27,28,29].

The policy for organ allocation was already detailed elsewhere [30,31].

All potential HCC candidates routinely underwent imaging re-assessment (tumor re-staging) approximately every 3 months; therefore, their priority was clinically re-assessed based on new radiological findings, liver function, and general clinical conditions. The present study was designed to evaluate the prognostic prediction of the TT staging system [15,16] as summarized in Figure 1. The re-staging time (ReS-time) used in the present study corresponded to the last available prior to LT or removal from the waiting-list (drop-out). At this landmark temporal endpoint, patients were classified into one of the following classes: TT0_C_, no residual tumor after curative treatment of HCC; TT0_L_, no residual tumor after locoregional embolo-therapies for transplantable HCC; TT1, single HCC ≤2 cm; TT0_NT_, no residual tumor after treatment of a non-transplantable HCC (successful downstaging); TT_FR_, transplantable HCC >T1 at first presentation or recurrent HCC >2 years after curative treatment; TT_UT_, transplantable HCC judged untreatable for reasons not captured by MELD (i.e., ascites); TT_PR_, partial response after complete bridge therapy in a transplantable tumor; TT_DR_, transplant eligibility after downstaging (sustained partial response) or recurrent HCC <2 years after curative treatment of any HCC. The 8 classes described were combined into three larger classes based on clinical evaluation of the tumor progression risk before LT and tumor recurrence risk after LT:

- High-risk-class (HRC) = stages TT_DR_ and TT_PR_;
- Intermediate-risk-class (IRC) = stages TT0_NT_, TT_FR_, and TT_UT_;
- Low-risk-class (LRC) = stages TT1, TT0_L_, and TT0.

### Statistical Analysis

Continuous data are reported as median and interquartile ranges (IQR) and categorical data as counts and percentages (%). Associations between continuous or categorical variables were evaluated using Wilcoxon test and Chi-square test, respectively. Survival functions were estimated using the Kaplan–Meier method. The cumulative incidences of being transplanted and of HCC progression without being transplanted were calculated from the time of restaging until the considered event. Drop-out from the waiting list due to other causes than HCC progression was considered as a competing event.

In transplanted patients, the cumulative incidence of HCC recurrence was calculated from the time of transplant until the evidence of recurrence. Death for causes unrelated to HCC was considered as a competing event, while patients alive and recurrence-free at time of the analysis were censored. Cumulative incidence functions were estimated according to the method described by Kalbfleisch and Prentice [32]. The Gray’s test [33] was used to assess cumulative incidence differences between groups.

Univariable and multivariable Fine and Gray proportional-hazards models [34], taking the competing risk of death for causes unrelated to HCC into account, were used to evaluate the effects on HCC recurrence of the time elapsed from the date of the last ReS and LT (categorized as less than two months and greater than or equal two months) and of the following variables evaluated at last ReS time: risk class (low, intermediate, or high), age, sex, AFP (ng/mL), number of nodules, diameter of the largest nodule, MELD score, tumor classification. The “2 months” cutoff has been choose basing on the median interval resulted between ReS-time and LT-time and basing on the general time needed to perform a living donor LT since the listing time [22]. In the final multivariable model, the number of nodules, the diameter of the largest nodule and the tumor classification at last ReS were not considered as independent variables because of their association with the risk classification assigned at the ReS-time. A *P* value <0.05 was considered statistically significant for all analyses. All the analyses were performed with the use of SAS software, version 9.4 (SAS Institute, Cary, North Carolina, USA).

## 5. Conclusions

In conclusion, the comprehensive assessment of TT stage seems to be effective in classifying patients into different groups and predicting tumor recurrence rates after transplant. The drop-out risk of patients evaluated at ReS-time seems to be proportional to the risk class assigned, moreover HRC is an independent risk factor for higher tumor recurrence rates after transplant. While a shorter time-to-transplant (< or ≥2 months after ReS-time) may play a favorable role for HRC, it negatively affects IRC patients. The IRC class needs to be explored much deeper, specially concerning differences between treated and untreated candidates’ stages. Our results suggest that physicians need to treat patients as much as possible before the ReS-time and to give them a priority based on the different TT stages. Finally, LDLT, when available, may guarantee a shorter and safer track for HRC. Further validation may be needed to reinforce these findings.

## Figures and Tables

**Table 1:** Listed patients’ characteristics (*N* = 376).

		Transplanted PTs ^1^ (*N* = 330)	Dropped-Out PTs ^2^ (*N* = 46)	Total PTs (*N* = 376)
Characteristics of PTs				
Age, median (IQR)		57 (52–61)	57 (52–60)	57 (52–61)
Sex, N (%)	Men	289 (88)	38 (83)	327 (87.0)
	Women	41 (12)	8 (17)	49 (13.0)
Presence of HCV, N (%)		205 (62)	31 (67)	236 (62.8)
Presence of HBV, N (%)		79 (24)	11 (24)	90 (23.9)
Abuse of alcohol, N (%)		95 (29)	8 (17)	103 (27.4)
Other cause of cirrhosis, N (%) ^3^		17 (5)	2 (4)	19 (5.1)
Reason for drop-out, N (%)	HCC progression		27 (59)	27 (59)
	Other ^4^		19 (41)	19 (41)
Parameter at the last restaging				
Restaging, N (%)	TT0_C_	6 (2)	2 (4)	8 (2.1)
	TT0_L_	93 (28)	11 (24)	104 (27.7)
	TT1	9 (3)	3 (6)	12 (3.2)
	TT0_NT_	15 (4)	0 (0)	15 (4.0)
	TT_FR_	32 (10)	7 (15)	39 (10.4)
	TT_UT_	16 (5)	0 (0)	16 (4.3)
	TT_PR_	80 (24)	10 (22)	90 (23.9)
	TT_DR_	79 (24)	13 (28)	92 (24.5)
AFP (ng/mL), median (IQR)		8 (3–35)	35 (5–208)	9 (3–43)
*Missing*		31	21	52
Number of nodules, median (IQR)		1 (0–2)	3 (1–5)	1 (0–2)
*Missing*		25	22	47
Diameter of the largest nodule (mm), median (IQR)		17 (14–23)	30 (14–45)	18 (14–25)
*Missing*		19	3	22
Time from the last TRT to restaging (days), median (IQR)		92 (44–245)	97 (37–294)	92 (42–257)
*Missing*		14	0	14
MELD score, N (%)	≤9	105 (32)	3 (18)	108 (31.4)
	10–19	193 (60)	10 (59)	203 (58.8)
	20–29	25 (7)	3 (18)	28 (8.1)
	≥30	3 (1)	1 (5)	6 (1.7)
	*Missing*	2	29	31
CHILD score, N (%)	A	152 (51)	4 (36)	156 (50.5)
	B	112 (38)	3 (27)	115 (37.2)
	C	34 (11)	4 (36)	38 (12.3)
	*Missing*	32	35	67
Tumor classification, N (%)	T0	103 (35)	3 (14)	106 (33.7)
	T1–T2	160 (55)	7 (32)	167 (53.0)
	T3–T4	30 (10)	12 (54)	42 (13.3)
	*Missing*	37	24	61
TRT, N (%)	Yes	273 (83)	42 (91)	315 (83.8)
Parameters and pathology at LT				
AFP (ng/mL), median (IQR)		8 (4–24)		8 (4–24)
*Missing*		35		35
Number of nodules, median (IQR)		2 (1–3)		2 (1–3)
Diameter of the largest nodule (mm), median (IQR)		20 (15–30)		20 (15–30)
Grade of HCC, N (%)	0	36 (12)		36 (12)
	1	34 (11)		34 (11)
	2	148 (48)		148 (48)
	3	93 (29)		93 (29)
	*Missing*	15		15
Microvascular invasion, N (%)	No	218 (73)		218 (73)
	Yes	82 (27)		82 (27)
	*Missing*	30		30
Macrovascular invasion, N (%)	No	289 (97)		289 (97)
	Yes	9 (3)		9 (3)
	*Missing*	32		32

PTs: patients, IQR: interquartile range, DG: diagnosis, LT: transplant. ^1^ Low risk: 108, intermediate risk: 63, high risk: 159. ^2^ Low risk: 16, intermediate risk: 7, high risk: 23. ^3^ Other causes of cirrhosis include: CRIPTO, HDV, HIV, emacromatosis, Wilson disease, sclerosing cholangitis, cholangitis primary biliary and secondary biliary cholangitis; ^4^ other cancers, chronic diseases, spontaneous regression of HCC.

**Table 2:** Association between patient or tumor characteristics and the risk of HCC recurrence after LT. (*N* = 330). Hazard ratios estimated using Fine and Gray proportional hazards models, considering death w/o HCC as first event as a competitive event.

	Univariate Model		Multivariate Model	
	HR (95% CI)	*P*	HR (95% CI)	*P*
Priority				
Low risk	Ref.		Ref.	
Intermediate risk	1.23 (0.47–3.19)	0.67	1.56 (0.51–4.74)	0.43
High risk	2.60 (1.28–5.26)	0.0079	2.89 (1.26–6.64)	0.012
Age (years)				
+10 years increase	0.91 (0.57–1.43)	0.67	0.93 (0.57–1.51)	0.77
Sex				
Women	Ref.		Ref.	
Men	0.90 (0.41–2.01)	0.80	0.93 (0.49–1.76)	0.81
AFP at the last restaging (ng/mL)				
Doubling the AFP value	1.22 (1.10–1.35)	<0.0001	1.23 (1.10–1.37)	0.0002
Number of nodules at the last restaging				
+1 nodule	1.24 (1.04–1.9)	0.018		
Diameter of the largest nodule at the last restaging (mm)				
+1 mm increase	1.03 (1.00–1.06)	0.026		
MELD score at the last restaging				
+1 pt increase	0.95 (0.89–1.02)	0.19	0.98 (0.90–1.07)	0.72
Tumor classification at the last restaging				
T0	Ref.			
T1–T2	1.79 (0.89–3.59)	0.10		
T3–T4	4.26 (1.83–9.96)	0.0008		
Time to LT from the last restaging				
<2 months	Ref.		Ref.	
≥2 months	1.25 (0.71–2.22)	0.44	1.25 (0.66–2.38)	0.50

HR: hazard ratio, LT: transplant, Ref.: reference category.

**Table 3:** Comparison between intermediate and high-risk patients transplanted within 2 months from restaging and after 2 months from restaging.

	Intermediate Risk		*P* ^1^	High Risk		*P* ^1^
	<2 months (*N* = 22)	≥2 months (*N* = 41)		<2 months (*N* = 78)	≥2 months (*N* = 81)	
AFP at LT, median (IQR)	12 (3–32)	6 (4–12)	0.24	9 (5–25)	11 (4–39)	0.43
Number of nodules, median (IQR)	2 (1–4)	2 (1–3)	0.73	2 (1–3)	2 (1–4)	0.32
Diameter of the largest nodule, median (IQR)	20 (15–28)	26 (20–35)	0.04	25 (15–30)	25 (18–30)	0.45
Macrovascular invasion, N (%)						0.49
No	21 (100)	36 (100)		72 (96)	72 (94)	
Yes	0 (0)	0 (0)		3 (4)	5 (6)	
Microvascular invasion, N (%)			0.85			0.35
No	16 (80)	28 (78)		50 (65)	45 (58)	
Yes	4 (20)	8 (22)		27 (35)	33 (42)	
Grading, N (%)			0.36			0.87
0	1 (5)	2 (5)		5 (6)	3 (4)	
1	4 (19)	4 (10)		5 (6)	6 (7)	
2	14 (67)	22 (57)		38 (50)	39 (49)	
3	2 (9)	11 (28)		29 (38)	32 (40)	

IQR: interquartile range, LT: transplant. ^1^ Wilcoxon test’s *P*-value for continuous variables, Chi-square test’s *P* for categorical variables.

## Supplementary Materials

The following are available online at https://www.mdpi.com/2072-6694/11/6/741/s1, Figure S1: Cumulative incidence of HCC recurrence after liver transplantation for all transplanted patients, stratified between patients without HBV and HCV virus (*N* = 61), patients with HBV virus (*N* = 64) and patients with HCV or both viruses (*N* = 205). Figure S2: (A) Intention-to-treat survival and (B) cumulative incidence of HCC recurrence or progression from listing (*N* = 376). Table S1: Transplanted patients’ characteristics (*N* = 330).

## References

[1] Fan H.L., Chen T.W., Hsieh C.B., Jan H.C., His S.C., De-Chuan C., Chu C.H., Yu J.C. (2010). Liver transplantation is an alternative treatment of hepatocellular carcinoma beyond the Milan criteria. Am. J. Surg..

[2] Lai Q., Vitale A., Iesari S., Finkenstedt A., Mennini G., Spoletini G., Hoppe-Lotichius M., Vennarecci G., Manzia T.M., Nicolini D. (2017). Intention-to-treat survival benefit of liver transplantation in patients with hepatocellular cancer. Hepatology.

[3] Forner A., Llovet J.M., Bruix J. (2012). Hepatocellular carcinoma. Lancet.

[4] Altekruse S.F., McGlynn K.A., Dickie L.A., Kleiner D.E. (2012). Hepatocellular carcinoma confirmation, treatment, and survival in surveillance, epidemiology, and end results registries, 1992–2008. Hepatology.

[5] Llovet J., Brú C., Bruix J. (1999). Prognosis of hepatocellular carcinoma: The BCLC staging classification. Semin. Liver Dis..

[6] Mazzaferro V., Regalia E., Doci R., Andreola S., Pulvirenti A., Bozzetti F., Montalto F., Ammatuna M., Morabito A., Gennari L. (1996). Liver transplantation for the treatment of small hepatocellular carcinomas in patients with cirrhosis. N. Engl. J. Med..

[7] Yao F. (2001). Liver transplantation for hepatocellular carcinoma: Expansion of the tumor size limits does not adversely impact survival. Hepatology.

[8] Yao F.Y., Mehta N., Flemming J., Dodge J., Hameed B., Fix O., Hirose R., Fidelman N., Kerlan R.K., Roberts J.P. (2015). Downstaging of hepatocellular cancer before liver transplant: Long-term outcome compared to tumors within Milan criteria. Hepatology.

[9] Mazzaferro V., Llovet J.M., Miceli R., Bhoori S., Schiavo M., Mariani L., Camerini T., Roayaie S., Schwartz M.E., Grazi G.L. (2009). Predicting survival after liver transplantation in patients with hepatocellular carcinoma beyond the Milan criteria: A retrospective, exploratory analysis. Lancet Oncol..

[10] Mazzaferro V., Sposito C., Zhou J., Pinna A.D., De Carlis L., Fan J., Cescon M., Di Sandro S., Yi-Feng H., Lauterio A. (2018). Metroticket 2.0 Model for analysis of competing risks of death after liver transplantation for hepatocellular carcinoma. Gastroenterology.

[11] Sharma P., Schaubel D.E., Guidinger M.K., Goodrich N.P., Ojo A.O., Merion R.M. (2011). Impact of MELD-based allocation on end-stage renal disease after liver transplantation. Am. J. Transplant..

[12] Vitale A., Volk M.L., De Feo T.M., Burra P., Frigo A.C., Ramirez Morales R., De Carlis L., Belli L., Colledan M., Fagiuoli S. (2014). A method for establishing allocation equity among patients with and without hepatocellular carcinoma on a common liver transplant waiting list. J. Hepatol..

[13] Toso C., Mazzaferro V., Bruix J., Freeman R., Mentha G., Majno P. (2014). Toward a better liver graft allocation that accounts for candidates with and without hepatocellular carcinoma. Am. J. Transplant..

[14] De Carlis L., Di Sandro S., Giacomoni A., Slim A., Lauterio A., Mangoni I., Mihaylov P., Pirotta V., Aseni P., Rampoldi A. (2012). Beyond the Milan criteria: What risks for patients with hepatocellular carcinoma progression before liver transplantation?. J. Clin. Gastroenterol..

[15] Mazzaferro V. (2016). Squaring the circle of selection and allocation in liver transplantation for HCC: An adaptive approach. Hepatology.

[16] Cillo U., Burra P., Mazzaferro V., Belli L., Pinna A.D., Spada M., Nanni Costa A., Toniutto P., on behalf of the I-BELT (Italian Board of Experts in the Field of Liver Transplantation) (2015). A multistep, consensus-based approach to organ allocation in liver transplantation: Toward a “Blended principle model”. Am. J. Transplant..

[17] Lai Q., Avolio A.W., Graziadei I., Otto G., Rossi M., Tisone G., Goffette P., Vogel W., Pitton M.B., Lerut J. (2013). Alpha-fetoprotein and modified response evaluation criteria in solid tumors progression after locoregional therapy as predictors of hepatocellular cancer recurrence and death after transplantation. Liver Transpl..

[18] Otto G., Schuchmann M., Hoppe-Lotichius M., Heise M., Weinmann A., Hansen T., Pitton M.P. (2013). How to decide about liver transplantation in patients with hepatocellular carcinoma: Size and number of lesions or response to TACE?. J. Hepatol..

[19] European Association For The Study Of The Liver, European Organisation For Research And Treatment Of Cancer (2012). EASL-EORTC clinical practice guidelines: Management of hepatocellular carcinoma. J. Hepatol..

[20] Salvalaggio P.R., Felga G., Axelrod D.A., Della Guardia B., Almeida M.D., Rezende M.B. (2015). List and liver transplant survival according to waiting time in patients with hepatocellular carcinoma. Am. J. Transplant..

[21] Di Sandro S., Slim A.O., Giacomoni A., Lauterio A., Mangoni I., Aseni P., Pirotta V., Aldumour A., Mihaylov P., De Carlis L. (2009). Living donor liver transplantation for hepatocellular carcinoma: Long-term results compared with deceased donor liver transplantation. Transplant. Proc..

[22] Halazun K.J., Patzer R.E., Rana A.A., Verna E.C., Griesemer A.D., Parsons R.F., Samstein B., Guarrera J.V., Kato T., Brown R.S. (2014). Standing the test of time: outcomes of a decade of prioritizing patients with hepatocellular carcinoma, results of the UNOS natural geographic experiment. Hepatology.

[23] Kulik L.M., Fisher R.A., Rodrigo D.R., Brown R.S., Freise C.E., Shaked A., Everhart J.E., Everson G.T., Hong J.C., Hayashi P.H. (2012). Outcomes of living and deceased donor liver transplant recipients with hepatocellular carcinoma: Results of the A2ALL cohort. Am. J. Transplant..

[24] Lo C.M., Fan S.T., Liu C.L., Chan S.C., Ng I.O.L., Wong J. (2007). Living donor versus deceased donor liver transplantation for early irresectable hepatocellular carcinoma. Br. J. Surg..

[25] Malagó M., Sotiropoulos G.C., Nadalin S., Valentin-Gamazo C., Paul A., Lang H., Radtke A., Saner F., Molmenti E., Beckebaum S. (2006). Living donor liver transplantation for hepatocellular carcinoma: A single-center preliminary report. Liver Transpl..

[26] Samoylova M.L., Dodge J.L., Yao F.Y., Roberts J.P. (2014). Time to transplantation as a predictor of hepatocellular carcinoma recurrence after liver transplantation. Liver Transpl..

[27] Choi H., Charnsangavej C., Faria S.C., Macapinlac H.A., Burgess M.A., Patel S.R., Chen L.L., Podoloff D.A., Benjamin R.S. (2007). Correlation of computed tomography and positron emission tomography in patients with metastatic gastrointestinal stromal tumor treated at a single institution with imatinib mesylate: Proposal of new computed tomography response criteria. J. Clin. Oncol..

[28] Eisenhauer E.A., Therasse P., Bogaerts J., Schwartz L.H., Sargent D., Ford R., Dancey J., Arbuck S., Gwyther S., Mooney M. (2009). New response evaluation criteria in solid tumours: Revised RECIST guideline (version 1.1). Eur. J. Cancer.

[29] Riaz A., Memon K., Miller F.H., Nikolaidis P., Kulik L.M., Lewandowski R.J., Ryu R.K., Sato K.T., Gates V.L., Mulcahy M.F. (2011). Role of the EASL, RECIST, and WHO response guidelines alone or in combination for hepatocellular carcinoma: Radiologic-pathologic correlation. J. Hepatol..

[30] De Carlis L., Di Sandro S., Centonze L., Lauterio A., Buscemi V., De Carlis R., Ferla F., Sguinzi R., Okolicsanyi S., Belli L. (2016). Liver-allocation policies for patients affected by HCC in Europe. Curr. Transplant. Rep..

[31] Wald C., Russo M.W., Heimbach J.K., Hussain H.K., Pomfret E.A., Bruix J. (2013). New OPTN/UNOS policy for liver transplant allocation: Standardization of liver imaging, diagnosis, classification, and reporting of hepatocellular carcinoma. Radiology.

[32] Prentice R.L., Kalbfleisch J.D., Peterson A.V., Flournoy N., Farewell V.T., Breslow N.E. (1978). The analysis of failure times in the presence of competing risks. Biometrics.

[33] Gray R.J. (1988). A Class of K-sample tests for comparing the cumulative incidence of a competing risk. Ann. Stat..

[34] Fine J.P., Gray R.J. (1999). A proportional hazards model for the subdistribution of a competing risk. J. Am. Stat. Assoc..

