# Supplementary material for: From a Philosophical Framework to a Valid Prognostic Staging System of the New “Comprehensive Assessment” for Transplantable Hepatocellular Carcinoma
Source: Cancers (Basel). 2019 May 28;11(6):741. doi: 10.3390/cancers11060741 (PMC6627952; doi:10.3390/cancers11060741)
Supplement: Supplementary file 1 [file cancers-11-00741-s001.pdf]

cancers

From a Philosophical Framework to a Valid Prognostic Staging System of the New "Comprehensive Assessment" for Transplantable Hepatocellular Carcinoma

Stefano Di Sandro, Vincenzo Bagnardi, Alessandro Cucchetti, Andrea Lauterio, Riccardo De Carlis, Laura Benuzzi, Maria Danieli, Francesca Botta, Leonardo Centonze, Marc Najjar and Luciano De Carlis MD

Table S1. Transplanted patients' characteristics (N = 330).

		Transplanted PTs			
		Low Risk (N = 108)	Intermediate Risk (N = 63)	High Risk (N = 159)	*P* [1]
Characteristics of PTs					
Age, median (IQR)		57 (52–61)	57 (53–61)	57 (52–62)	0.80
Sex, N (%)	Men	91 (84)	52 (83)	146 (92)	0.07
	Women	17 (16)	11 (17)	13 (8)	
Presence of HCV, N (%)	No	47 (44)	23 (37)	55 (35)	0.33
	Yes	61 (56)	40 (63)	104 (65)	
Presence of HBV, N (%)	No	79 (73)	48 (76)	124 (78)	0.66
	Yes	29 (27)	15 (24)	35 (22)	
Abuse of alcohol, N (%)	No	74 (69)	44 (70)	117 (74)	0.64
	Yes	34 (31)	19 (30)	42 (26)	
Other cause of cirrhosis, N (%) [2]	No	105 (97)	59 (94)	149 (94)	0.40
	Yes	3 (3)	4 (6)	10 (6)	
Parameters at listing					
AFP (ng/mL), median (IQR)		8 (5–20)	8 (4–14)	9 (4–25)	0.31
Missing		6	1	12	
Number of nodules, median (IQR)		1 (1–1)	2 (1–2)	1 (1–2)	<0.0001
Diameter of the largest nodule (mm), median (IQR)		20 (15–27)	23 (16–35)	24 (16–30)	0.04
Missing		6	3	7	
MELD score, N (%)	≤9	34 (32)	15 (25)	61 (39)	0.0005
	10–19	67 (62)	35 (57)	91 (59)	
	20–29	4 (4)	10 (16)	3 (2)	
	≥30	2 (2)	1 (2)	0 (0)	
	Missing	1	2	4	
CHILD score, N (%)	A	46 (47)	17 (31)	92 (62)	<0.0001
	B	39 (40)	21 (39)	51 (34)	
	C	13 (13)	16 (30)	6 (4)	
	Missing	10	9	10	
Macrovascular invasion, N (%)	No	102 (98)	57 (100)	146 (98)	0.56
	Yes	2 (2)	0 (0)	3 (2)	
	Missing	4	6	10	
Tumor classification, N (%)	T1–T2	102 (100)	43 (72)	119 (78)	<0.0001
	T3–T4	0 (0)	17 (28)	33 (21)	
	Missing	6	3	7	
Parameter at the last restaging					
Restaging, N (%)	$TT0_C$	6 (5.6)			
	$TT0_L$	93 (86.1)			

		Transplanted PTs			P [1]
		Low Risk (N = 108)	Intermediate Risk (N = 63)	High Risk (N = 159)	
	TT1	9 (8.3)	-	-	
	$TT0_{NT}$		15 (23.8)		
	TT_{FR}		32 (50.8)		
	TT_{UT}		16 (25.4)		
	TT_{PR}			80 (50.3)	
	TT_{DR}			79 (49.7)	
AFP (ng/mL), median (IQR)		8 (3–36)	8 (4–17)	8 (3–39)	0.98
Missing		9	3	19	
Number of nodules, median (IQR)		0 (0–0)	1 (0–2)	2 (1–3)	<0.0001
Missing		4	8	13	
Diameter of the largest nodule (mm), median (IQR)		15 (12–17)	21 (16–28)	17 (14–21)	0.02
Missing		1	5	13	
Time from the last TRT to restaging (days), median (IQR)		85 (44–217)	67 (5–177)	115 (44–260)	0.52
Missing		3	0	11	
MELD score, N (%)	≤9	34 (32)	20 (32)	51 (32)	0.0014
	10–19	66 (61)	29 (46)	98 (62)	
	20–29	4 (4)	13 (21)	8 (5)	
	≥30	3 (3)	1 (2)	1 (1)	
	Missing	1	0	1	
CHILD score, N (%)	A	46 (47)	17 (31)	89 (61)	<0.0001
	B	39 (40)	21 (39)	52 (36)	
	C	13 (13)	16 (30)	5 (3)	
	Missing	10	9	13	
Tumor classification, N (%)	T0	88 (85)	15 (29)	0 (0)	<0.0001
	T1-T2	15 (14)	32 (63)	113 (82)	
	T3-T4	1 (1)	4 (8)	25 (18)	
	Missing	4	12	21	
TRT, N (%)	No	9 (8)	48 (76)	0 (0)	<0.0001
	Yes	99 (92)	15 (24)	159 (100)	
Parameters and pathology at LT					
AFP (ng/mL), median (IQR)		7 (4–23)	7 (4–17)	10 (5–34)	0.05
Missing		13	3	19	
Number of nodules, median (IQR)		1 (0–2)	2 (1–3)	2 (1–3)	<0.0001
Diameter of the largest nodule (mm), median (IQR)		16 (10–20)	25 (15–35)	25 (15–30)	<0.0001
Grade of HCC, N (%)	0	25 (27)	3 (5)	8 (5)	<0.0001
	1	15 (16)	8 (13)	11 (7)	
	2	35 (37)	36 (60)	77 (49)	
	3	19 (20)	13 (22)	61 (39)	
	Missing	11	3	1	
Microvascular invasion, N (%)	No	79 (89)	44 (79)	95 (61)	<0.0001
	Yes	10 (11)	12 (21)	60 (39)	
	Missing	19	7	4	
Macrovascular invasion, N (%)	No	88 (99)	57 (100)	144 (95)	0.06
	Yes	1 (1)	0 (0)	8 (5)	
	Missing	19	6	7	

PTs: patients, IQ: interquartile range, DG: diagnosis, LT: transplant. [1] Chi-square P for categorical variables, Wilcoxon P for continuous variables. [2] Other causes of cirrhosis include: CRIPTO, HDV, HIV, emacromatosis, Wilson disease, sclerosing cholangitis, cholangitis primary biliary, and secondary biliary cholangitis.

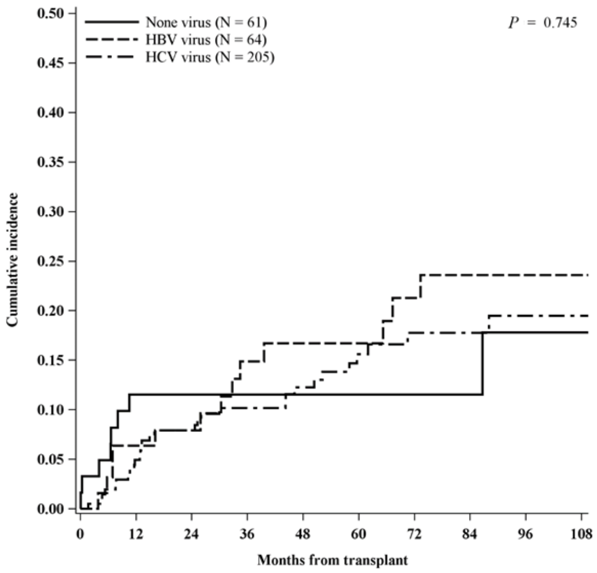

Figure S1. Cumulative incidence of HCC recurrence after liver transplantation for all transplanted patients, stratified between patients without HBV and HCV virus (N = 61), patients with HBV virus (N = 64), and patients with HCV or both viruses (N = 205).

(A)

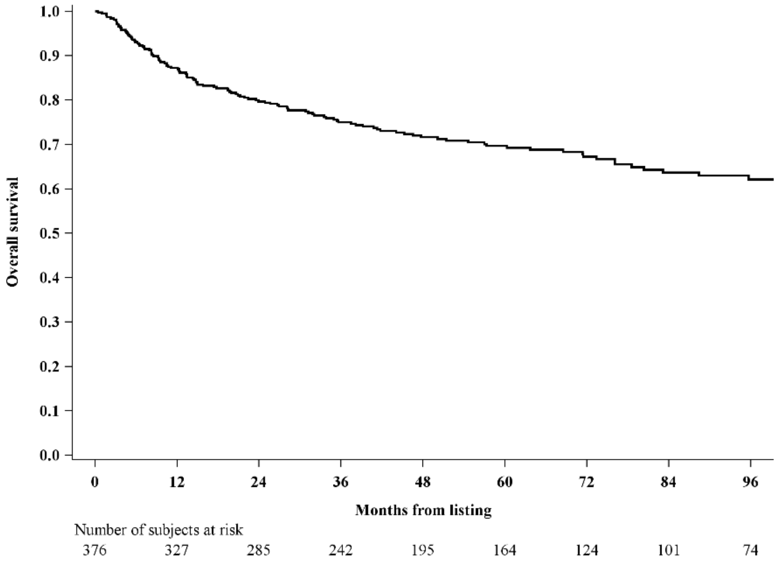

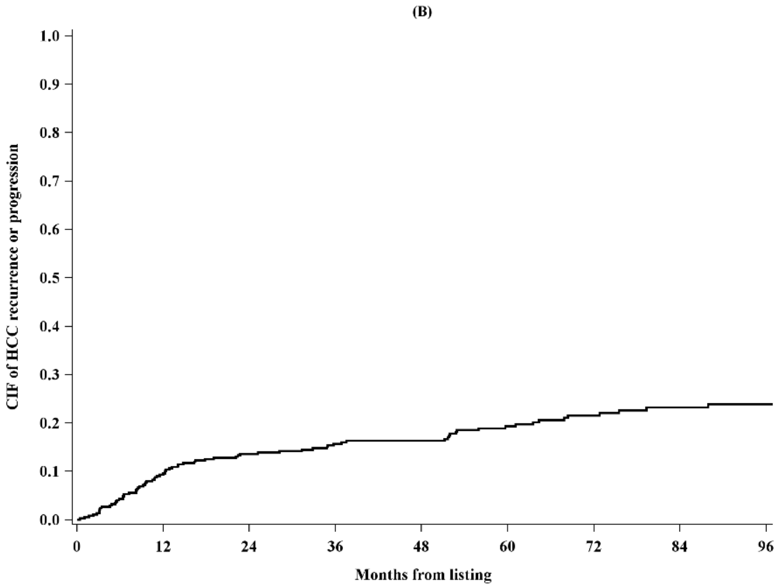

Figure S2. (**A**) Intention-to-treat survival and (**B**) cumulative incidence of HCC recurrence or progression from listing (N = 376).
